# An Untargeted Metabolomics Approach to Study the Variation between Wild and Cultivated Soybeans

**DOI:** 10.3390/molecules28145507

**Published:** 2023-07-19

**Authors:** Fakir Shahidullah Tareq, Raghavendhar R. Kotha, Savithiry Natarajan, Jianghao Sun, Devanand L. Luthria

**Affiliations:** 1 Methods and Application of Food Composition Laboratory, Beltsville Human Nutrition Research Center, Agricultural Research Service, U.S. Department of Agriculture, Beltsville, MD 20705, USA; fakir.tareq@usda.gov (F.S.T.); raghavendhar.kotha@usda.gov (R.R.K.); jianghao.sun@usda.gov (J.S.); 2 Soybean Genomics and Improvement Laboratory, Beltsville Agricultural Research Center, Agricultural Research Service, U.S. Department of Agriculture, Beltsville, MD 20705, USA

**Keywords:** soybean cultivars, metabolomics, ultra-high-performance liquid chromatography-tandem mass spectrometry, multivariate analysis

## Abstract

The differential metabolite profiles of four wild and ten cultivated soybeans genotypes were explored using an untargeted metabolomics approach. Ground soybean seed samples were extracted with methanol and water, and metabolic features were obtained using ultra-high-performance liquid chromatography coupled with high-resolution mass spectrometry (UHPLC-HRMS) in both positive and negative ion modes. The UHPLC-HRMS analysis of the two different extracts resulted in the putative identification of 98 metabolites belonging to several classes of phytochemicals, including isoflavones, organic acids, lipids, sugars, amino acids, saponins, and other compounds. The metabolic profile was significantly impacted by the polarity of the extraction solvent. Multivariate analysis showed a clear difference between wild and cultivated soybean cultivars. Unsupervised and supervised learning algorithms were applied to mine the generated data and to pinpoint metabolites differentiating wild and cultivated soybeans. The key identified metabolites differentiating wild and cultivated soybeans were isoflavonoids, free amino acids, and fatty acids. Catechin analogs, cynaroside, hydroxylated unsaturated fatty acid derivatives, amino acid, and uridine diphosphate-*N*-acetylglucosamine were upregulated in the methanol extract of wild soybeans. In contrast, isoflavonoids and other minor compounds were downregulated in the same soybean extract. This metabolic information will benefit breeders and biotechnology professionals to develop value-added soybeans with improved quality traits.

## 1. Introduction

Soybean is an important leguminous crop providing meal and oil for food (~20%), animal feed (~76%), biodiesel, and other industrial uses (~4%) [1]. According to an estimation by the U.S. Department of Agriculture, in the 2019/2020 harvest year, the world soybean production was 337 million tons, and an increase of 8% (362 million tons) was observed in the 2020/2021 harvest year [2]. Three countries, namely Brazil, the United States of America, and Argentina, accounted for approximately 80% of the world’s production of soybeans [3]. In the United States, the total soybean production was 4.3 billion bushels in 2022 [4]. Soybeans contain diverse primary and secondary metabolites, including proteins, carbohydrates, lipids, amino acids, and phytochemicals (isoflavones, saponins) that contribute to their nutritional and health-promoting properties [5]. Global breeding programs and biotechnology approaches have been used to develop new varieties of soybeans with improved quality traits. A better understanding of how agronomic practices, environmental conditions, pre-and post-harvest storage, and processing conditions impact metabolite changes in soybeans is essential to further develop soybeans with improved quality traits.

The metabolome of plants contains thousands of a diverse range of phytochemicals that can be broadly classified into three categories: primary metabolites (required for plant growth), secondary metabolites (mediate plant-environment interactions), and hormones (organismal processes and metabolism) [6]. Metabolites play an important role in a plant’s growth, development, and responses to environmental factors. Plant metabolomics has been applied to understand changes in the different stages of plant growth [7], changes in the metabolism of plants to environmental contaminants [8], impacts of soil types [9], and the effect of abiotic and biotic stresses [10,11,12].

Several targeted and untargeted metabolomic studies in soybean have been reported. Clarke et al. used a metabolomics approach to show that the metabolome of the genetically modified soybean line had no significant deviation from natural variation within the soybean metabolome, except for targeted changes in the metabolized bioengineered pathway [13]. In a separate study, Chebrolu et al. evaluated the impact of stress during seed development by exposing plants to optimum, moderate, or high temperatures. The authors showed that the germination of seeds from the heat-susceptible genotype is reduced by 50% for the 36/24 °C treatment and completely inhibited for the 42/26 °C. The same authors also showed the enrichment of tocopherols, flavonoids, phenylpropanoids, and ascorbate precursors in the seeds of the heat-tolerant genotype [14].

Gupta et al. used integrative proteomics and metabolomics analyses to identify the differentially expressed proteins and metabolites in two contrasting yellow (Mallikong) and brown-colored (Mallikong mutant) soybean seeds. The authors showed proteins involved in primary metabolism were downregulated in Mallikong mutant (MM), suggesting energy in MM might be utilized for proanthocyanidin biosynthesis responsible for the development of brown seed coat color [15]. Recently, Bragagnolo et al. using a metabolomics approach, showed the presence of isoflavonoids, flavonoids, terpenes, and other substances in soy stems, leaves, pods, and roots. The authors suggested that these underused parts of the plant can potentially be used as a source of bioactive compounds [16].

Studies on the effects of genotype and environment (temperature, carbon dioxide level, and water stress) on soybean isoflavone contents were also conducted [17,18]. The authors observed significant variations in total and individual isoflavone contents between genotype × year, genotype × location, and genotype × year × location interactions using a targeted metabolomic approach. Similarly, metabolic changes in soybean roots under water deficit stress were investigated by gas chromatography-mass spectrometry (GC-MS), and changes in several metabolic pathways involved in sugars, amino acids, and isoprenoid metabolisms were reported [19].

Previously, we reported metabolic responses of nine soybean varieties grown under field and greenhouse conditions. Soybean extracts were derivatized and assayed by GC-FID, GC-MS, and LC-MS to identify ten primary (amino acids, organic acids, and sugars) and ten secondary (isoflavones, fatty acid methyl esters) metabolites. We found that the free amino acids and organic acids varied between the varieties [20]. In a recent study, we reported the compositional analysis of fatty acids and soluble sugars in wild and cultivated 14 soybeans genotypes using targeted analysis [21]. We observed differences in total oil content in wild soybeans (~9%) versus cultivated soybeans (16–22%). In addition, higher levels of linolenic acid (~17%) and stachyose (~53%) were determined in the wild type. In contrast, higher levels of oleic acid (~19%) and sucrose (~59%) were detected in cultivated soybeans using ion chromatography with amperometry detection and gas chromatography with a flame ionization detector after transesterification of crude oil.

The above-targeted methodologies required detailed sample preparation, separation, and derivatization steps to identify and quantify a limited number of metabolites. Untargeted methods such as ultraviolet spectrophotometry (UV), infrared (IR), and near-infrared (NIR) spectrometry have also been used to acquire spectral fingerprints to evaluate sources of variances based on the classes of compounds. The objective of the current study was to investigate if the untargeted UHPLC-HRMS approach can be used to differentiate soybean cultivars and identify the metabolites responsible for the differentiation. Furthermore, we also wanted to evaluate the role of extraction solvents in untargeted metabolomics.

## 2. Results and Discussion

In this study, we selected four wild and ten cultivated soybean genotypes from different regions of the world for untargeted metabolomics investigation. Methanol and water extracts of all samples were analyzed to investigate their metabolic profiles in both positive and negative ion modes.

### 2.1. Identification of Compounds

Thousands of metabolite features were obtained using the Compound Discoverer 3.3 software program. Among them, 98 metabolites belonging to amino acids, organic acids, sugars, isoflavones, and soy saponins in methanol and water extracts were identified by careful analysis of the MS/MS fragments and comparison with the available literature data. A total of 64 compounds were detected with the positive and negative ion modes in the water extract, whereas 35 compounds were identified in the methanol extract. Some compounds were detected in both extracts (methanol and water) and in both ionization modes (positive and negative). The details of the compounds identified are summarized in Table 1 (methanol extract) and Table 2 (water extract).

Soybean seeds are one of the most concentrated natural sources of isoflavones in human diets [22]. In soybeans, isoflavones are found in free and conjugated forms. In conjugated forms, they can occur with sugars (glucosides) and/or acids (acetyl/malonyl). The three common free isoflavones in soybean seeds are genistein, daidzein, and glycitein. The [M + H]^+^ and [M − H]^−^ for daidzein, genistein, and glycitein were observed at *m*/*z* 255.0650, 271.0612, 285.0758, and 253.0506, 269.0454, 283.0611 respectively. In the present study, we identified five analogs of daidzein at t*_R_* 0.99, 5.82, 6.26, 6.58, and 8.23 min. The compounds can be conjugated with sugars, acetylated, and/or malonylated. There are several reports of the presence of such analogs in the literature during targeted analysis [23,24,25].

Amino acids were detected as the second major group of metabolites in soybean seeds. Soybean seeds provide a rich source of plant-based proteins. The protein content in soybean seeds is around 40%, as documented in the published literature [26,27]. We identified 17 free amino acids and two acetylated analogs of amino acids in the water extracts using positive and negative ion modes. Only six amino acids were detected in the methanol extracts. Similar results of the presence of amino acids from soybeans using targeted analysis after derivatization with aminopyridyl-*N*-hydroxysuccinimidyl carbamate (APDS) reagent [28] have been reported.

In addition to amino acids and isoflavones, over 60 other organic compounds were also detected in water and methanol extracts of soybean seeds. These include organic acids, flavonoids, sugars, saponins, fatty acids, and other phytochemicals. Organic acids are one of the major components affecting soybeans’ overall quality and taste. A recent study by Hyeon et al. reported some organic acids in soybean, such as malic acid, citric acid, and succinic acid [29]. In another recent study, ten organic acids were reported using NMR analysis by Song et al. in soybeans [30]. Similarly, the presence of epicatechin and sugars have also been reported previously by Hyeon et al. and Song et al. [29,30]. We also reported in our earlier publication the presence of sugars in soybean by ion chromatography and fatty acids after derivatization using GC-MS analysis with targeted analysis [20,21].

### 2.2. Comparison of Metabolites among Cultivars

**Figure 1:**
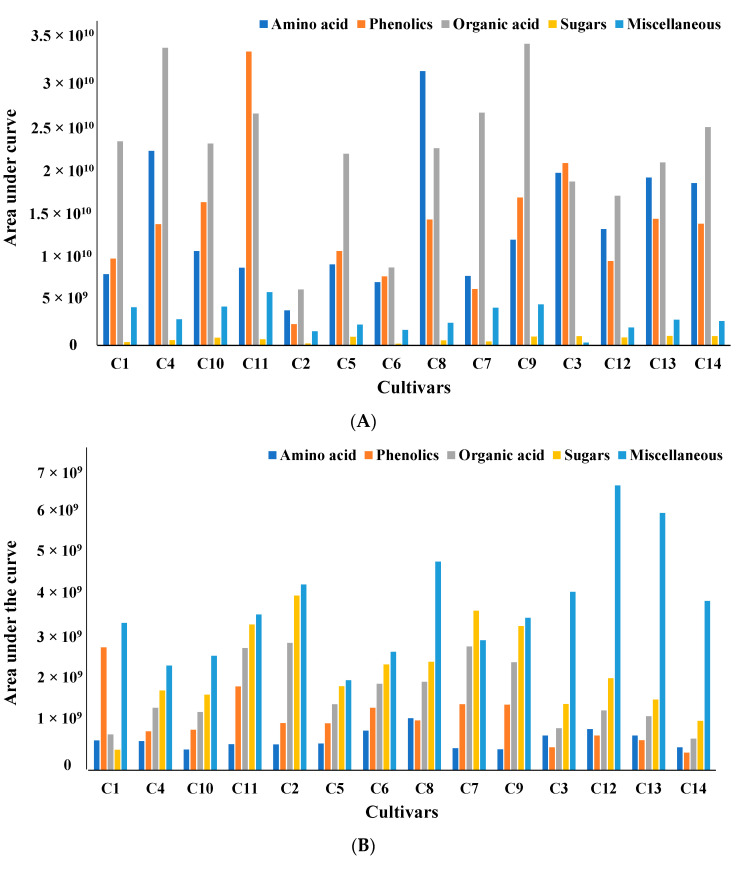
(**A**) Comparative analysis of all metabolites based on area under the curve for four wild and ten cultivated soybeans extracted with water. All samples were analyzed in triplicate, and the averages of the triplicates have been reported. (**B**) Comparative analysis of all metabolites based on area under the curve for four wild and ten cultivated soybeans extracted with methanol. All samples were analyzed in triplicate, and the averages of the triplicates have been reported.

All metabolites can be broadly classified into five major subgroups: amino acids, phenolics, organic acids, sugars, and miscellaneous. To compare the amounts of metabolites produced in each cultivar, all metabolites were organized based on the area under the curve for the mass ion extracted with two different solvents, water (Figure 1A) and methanol (Figure 1B).

The total amount of amino acids, one of the significant classes of metabolites obtained from water extract, varied significantly between 25% and 75% within cultivated and wild soybean cultivars. Based on the areas under the curve for the targeted mass ion maximum amount of amino acids was obtained in the Asian cultivar (C8). Assessments of protein quality of 14 soybean cultivars using targeted amino acid analysis and two-dimensional electrophoresis were investigated by Zarkadas et al. The authors indicated that all fourteen cultivars contained a good balance of essential amino acids [31]. Similar free amino acids were observed in fermented and unfermented soybeans and mung beans using targeted amino acid analysis after derivatization. The content of free amino acids was increased by 13-fold and 32-fold in fermented mung and soybeans, respectively. The authors showed that fermentation improved the amino acid content in a single soy and mung bean cultivar [32]. A similar analysis and characterization of the amino acid content of thua nao, a traditionally fermented food of northern Thailand, was studied by Dajanta et al. [33]. Significant variation in the phenolics area was seen in different cultivars. A maximum amount of phenolics was detected in the modern elite cultivar C11. The relative percentage of phenolics in other cultivars varied between 7% and 62%, with the lowest amount in cultivar C2. Similar variations in the total phenolic content (6.67 μg^−1^ in Pureunkong to 72.33 μg^−1^ in Poongsannamulkong) were observed in seven cultivars of soybeans by Kim et al. [34]. However, the maximum amount of organic acids were detected in ancestral (C9) and modern elite (C4) cultivars, with others showing variation between 25 and 77%.

However, compared to the metabolites from water extract, phenolics were the predominant metabolites in methanol extract. It has been documented that methanol, ethanol, acetone, water, and their water mixtures, with or without acids, are the most widely used solvents for extracting phenolic compounds [35,36]. Boeing et al. reported that among the pure solvents, methanol is the most effective solvent for the extraction of antioxidant compounds [37,38].

Significant variations of phenolics content were observed in the present study between cultivars (33–95%). The maximum amount of phenolics was detected in wild cultivar C13, with the lowest amount in Asian cultivar C8. This was different from the water extract, where the modern elite C11 cultivar showed the maximum amount. Similarly, the total amount of amino acid varied between 40 and 80% among cultivars. Similar to water extract maximum amount of amino acid was obtained in the Asian cultivated cultivar (C8) in methanol extract. However, the maximum amount of organic acid was detected in Asian (C2 and C5) cultivars, with others showing variation between 20 and 90%. The total amount of sugar varied between 30–90%, and Asian cultivar C8 produced the maximum amount of sugar compared to other cultivars. Since no distinct systematic variations in the area under the curve for the mass ions of different metabolites between cultivars were observed, multivariate analysis was done to differentiate between cultivars.

### 2.3. Classification of Wild and Cultivated Soybeans Using Principal Component Analysis (PCA) and Volcano Plots

**Figure 2:**
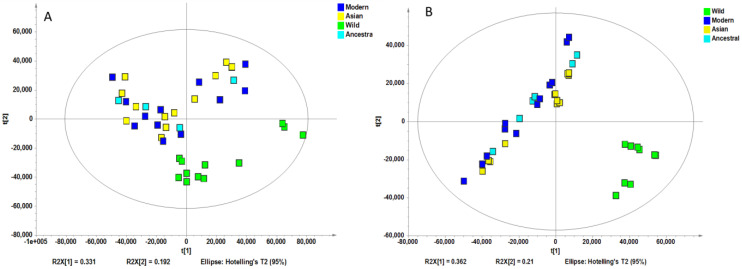
Principal component analysis of the high-resolution mass spectral data of all metabolites for four wild and ten cultivated soybeans extracted with methanol (**A**) and water (**B**).

**Figure 3:**
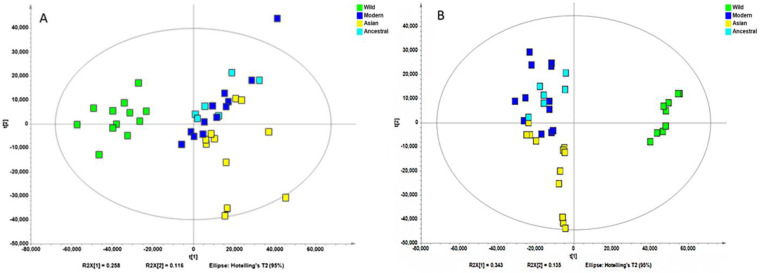
Partial least squares discriminate analysis (PLS-DA) of the high-resolution mass spectral data of all metabolites for four wild and ten cultivated soybeans extracted with methanol (**A**) and water (**B**).

Non-supervised analysis of the entire UHPLC-HRMS data using the Progenesis QI resulted in the detection of several thousands of ion features from each extract. The intensity of each ion was extracted and used for principal component analysis. PCA of the normalized intensity data of methanol and water extracts showed certain differentiation of wild and cultivated soybeans, as shown in Figure 2A and Figure 2B. The variances captured by the two components (PC1 and PC2) were between 29–49%. A further supervised partial least square discriminant analysis (PLS-DA) was performed, and the score plots are shown in Figure 3A and Figure 3B. Clear separations were observed between the wild and cultivated soybeans on the score plots; however, the separation between the cultivated soybeans was not obvious. For the metabolites from methanol extraction, the PLS-DA model resulted in the cross-validated predictive ability Q2(Y) of 39.9%. A value of 37.4% of the variance in X [R2(X)] was used to account for 21.1% of the variance of Y [R2(Y)]. For the metabolites from water extraction, the PLS-DA model resulted in the cross-validated predictive ability Q2 of 64.8%. A value of 84.2% of the variance in X [R2(X)] was used to account for 84.8% of the variance of Y [R2(Y)]. It suggested that the model from the metabolites from the water extraction gave us better prediction ability. The *t*-test (*p*-value) and fold change served as criteria for selecting the most discriminatory metabolites.

**Figure 4:**
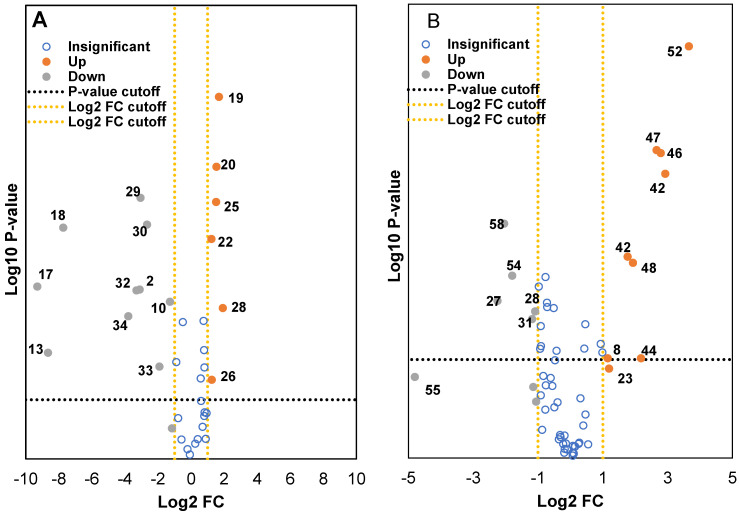
Volcano plots of identified metabolites from ten cultivated and four wild soybeans extracted with methanol (**A**) and water (**B**). The numbers noted in the score plot are marked as * in Table 1 for the methanol extract and Table 2 for the water extract.

Two volcano plots were constructed to identify the metabolites from both methanol and water extracts that were differentially expressed in wild and cultivated soybeans (Figure 4A and Figure 4B). Around 150–400 metabolite ion features with selected threshold fold change (≥4) and *t*-tests threshold (*p* ≤ 0.05) were selected as cutoff values for the volcano plots to identify the prominent ions responsible for the variation of metabolites. The data were categorized into three fractions: statistically insignificant (blue), upregulated (orangish-brown), and downregulated (grey). As seen with the filtered data set, which contained several hundreds of metabolites, a few compounds were either up or downregulated between wild and cultivated soybeans. Careful analysis of the fragmentation ions and comparison with the literature data significantly reduced the number of putatively identified compounds that were downregulated in cultivated and wild soybeans. Metabolites upregulated in the methanol extract of wild soybeans compared to the cultivated soybeans were catechin analog, cynaroside, hydroxylated unsaturated fatty acid derivatives, and uridine diphosphate-*N*-acetylglucosamine. Similar observations were also identified by Hyeon et al., where the authors showed with PCA that amino acids, organic acids, and fatty acids were higher in cultivated black soybeans as compared to wild black soybeans [30]. However, higher content of isoflavones and other flavonoids derivative was determined in the cultivated soybeans compared to wild soybeans. Some of the metabolites identified in the water extract showed similar trends. The isoflavones analogs, soy saponin, flavonoid analogs, amino acids (glutamine and guanine), lactic acid derivative, and 6-hydroxy caproic acid were determined in higher amounts in cultivated soybean as compared to wild soybeans. Upregulated compounds in wild soybeans were tentatively identified as phloretin, hypoxanthine, glutaric acid, and tyrosine. These results will be of significant value to soybean breeders and biotechnology researchers to develop new varieties of value-added soybeans with improved qualitative traits.

## 3. Materials and Methods

### 3.1. Solvents and Materials

LC-MS-grade solvents, including acetonitrile, methanol, water, and formic acid, were used for the extraction and chromatographic separation. These organic solvents were purchased from Fisher Scientific (Pittsburgh, PA, USA). Extractions were carried out in a 15 mL centrifuge tube obtained from Thermo Scientific (Waltham, MA, USA). Polyvinylidene difluoride (PVDF) syringe filters with a pore size of 0.45 µm were purchased from National Scientific Company (Duluth, GA, USA).

### 3.2. Samples

Fourteen soybean cultivars were collected for this study. Four of these cultivars are wild (C3, C12–C14), and the other ten cultivated cultivars are categorized into three groups, namely, Asian landraces (C2, C5, C6, and C8), ancestral (C7 and C9), and modern elite (C1, C4, C10, and C11). All soybean samples were obtained from the soybean germplasm collection (USDA, Urbana, IL, USA). The cultivar details, i.e., accession number, origin, and genotype information (wild soybean (*G. soja*), soybean bred for seed traits, and soybean landraces), were previously reported in our earlier publication [22]. In the present study, soybean seeds were ground in a commercial coffee grinder and stored in an ultralow temperature (<−60 °C) freezer prior to analysis.

### 3.3. Extraction of Metabolites

An amount of 150 ± 0.05 milligrams (mg) of soybean seed powder of each sample were taken into 15 mL centrifuge tubes and extracted with 5 mL of methanol (polarity index 5.1) in an ultrasonic bath (power 400 watts, Advanced Sonic Processing Systems, Oxford, CT, USA) for 15 min (twice). Similarly, the extraction of samples with water (polarity index 10.2) was also carried out. The extracts were centrifuged at 4000 rpm for 15 min and filtered using a 0.45 µm PVDF filter. The clean filtrate (500 µL) containing extracted metabolites was transferred to 2 mL HPLC vials and subjected to UHPLC-MS/MS analyses. All analyses were carried out in triplicate.

### 3.4. Data Acquisition

The Vanquish UHPLC system (Thermo Scientific), consisting of a binary pump, column compartment, autosampler, and detectors (Photodiode Array detector) PDA and (Charged aerosol detector) CAD coupled with an Exploris 240 mass spectrometer, was used to acquire high-resolution mass data in full-scan and data-dependent acquisition mode for all samples. An aliquot of each extract was analyzed using both positive and negative ionization modes. The metabolites were separated on a C_18_ Agilent column (Eclipse Plus, 4.5 × 50 mm, 1.8 µm, 1200 bar pressure limit) using the gradient programs; 10% B at 0 min, gradually moves to 30% at 5 min, reach 60% at 10 min, reach 95% at 15 min, run 95% at 15–18 min, then reduced to 10% at 18.5 min. Water and acetonitrile acidified with 0.1% formic acid were used as mobile phases A and B, respectively. The flow rate and the injection volume were maintained at 0.5 mL/min, and 10 µL, respectively.

The HRMS mass range was from 100–2000 *m*/*z*, and the ESI conditions were as follows; sheath gas, auxiliary, and sweep gas at 50, 10, and 1 (arbitrary units), respectively, spray voltage at 3.4 kV, and capillary temperature at 320 °C, and vaporizer temperature 350 °C. The full scan mass spectra and three DD-MS^2^ events were acquired at a resolving power of 12,000. An isolation width of 1 amu, maximum ion injection time of 100 ms, stepped collision energy starting from 30, 50, and 150, and an activation time of 10 ms was used for MS*^n^* activation. Xcalibur 4.4, including FreeStyle 1.8 software packages, has been used to analyze the mass spectral data.

### 3.5. Identification of Compounds

Compound Discoverer (Version 3.3, Thermo Scientific) software was used for the putative identification of metabolites. This involved application of several filters, namely background subtraction, MS*^n^* fragmentation information, ΔMass (±5 ppm), minimum area, Fish score, and screening data with multiple databases (in-house mass library for soybean, mzCloud, ChemSpider, Metabolika, and other online available databases available). Furthermore, mass spectral data (molecular ion mass (*m*/*z*) and MS/MS fragmentation patterns) were compared with literature-reported data [22,23,24].

### 3.6. Data Processing

Raw LC-MS data were analyzed using the Compound Discoverer program to collect features for statistical analysis. The overall workflow of the program includes the detection of chromatographic peaks, extraction of the MS spectrum, deconvolution of the overlapping ions based on their isotope patterns, and integration of their respective peak areas. Acquired UHPLC-HRMS raw files were processed by using Nonlinear Progenesis QI (Durham, NC, USA) for peak detection, noise filtering, and peak alignment. Important deconvolution parameters were mass tolerance of 5 ppm, retention time tolerance of 0.2 min, peak rating threshold of 4, minimum peak intensity of 10^9^, chromatographic threshold S/N of 1.5, CV contribution of 10, and an area contribution of 3. The resulting areas of each sample in triplicate were exported to Microsoft Excel 365 (Microsoft, Redmond, WA, USA) for volcano plot construction.

### 3.7. Statistical Analysis

A data matrix was generated from Progenesis Qi, including a variable index (paired *m*/*z*-retention time), sample names (observations), and peak intensities. The peak intensities in each sample were scaled by Pareto scaling before further multivariate analysis using SIMCA 13.0 (Sartorius Stedim Biotech, Umeå, Sweden). Key metabolites responsible for separating different soybean genotypes were further isolated by constructing volcano plots using the Microsoft Excel application. Based on the loadings scores and a threshold of 0.05 for the Student’s *t*-test of individual samples, key metabolites were selected and identified. The log_10_ value of the peak area was used to compare the levels of metabolites between samples.

## 4. Conclusions

Advances in technology, data collection, and analysis software allowed for easy differentiation of wild and cultivated soybean using UHPLC coupled with HRMS without any chemical derivatization in conjunction with PCA analysis. Several recent publications on metabolomics analysis in peer-reviewed journals often use a single aqueous alcohol solvent mixture for sample extraction. As plants produce hundreds and thousands of metabolites, it is critical to investigate multiple solvent compositions of varying polarity to optimize the extraction of a wide array of metabolites with varying polarity. In this manuscript, we showed that the metabolites extracted and putatively identified in two different solvents with polarity indexes of 5.1 and 10.2 were significantly different, with some overlapping metabolites. A total of 98 metabolites were putatively identified as isoflavones, organic acids, lipids, sugars, amino acids, saponins, and other compounds. The PCA and PLS-DA analysis of the HRMS data allowed easy classification of wild and cultivated soybeans. The major metabolites that allowed the differentiation of wild and cultivated soybeans were isoflavonoids, amino acids, and fatty acids. Several metabolites were up and downregulated in wild soybeans as compared to cultivated ones. In general, metabolites upregulated in the wild soybeans were catechin analogs, cynaroside, hydroxylated unsaturated fatty acid derivatives, amino acid, and uridine diphosphate-*N*-acetylglucosamine. Downregulated metabolites were identified as isoflavonoids and other minor compounds. These marker metabolites may link to characteristic performance traits desired in soybean breeding for crop improvement. In conclusion, the metabolomics extraction and workflow with solvents of varying polarity indexes can result in a significant increase in the number of metabolites extracted and identified. This will allow researchers to extract and identify multiple biomarkers that can provide insights into metabolic pathways and also increase our understanding of how plants interact with varying climatic and growth conditions. It will also enable researchers to breed plants sustainable to various abiotic and biotic stresses. In addition, the detailed metabolomics information will aid researchers in producing foods with better nutritional traits and yields. This will be needed to improve sustainable agriculture practices and alternatives to animal-based protein products that may potentially provide solutions for the global food security challenge with increasing global population and decreasing agricultural land acreages.

## Figures and Tables

**Table 1:** Identification of metabolites using high-resolution mass spectrometry of methanol extract of fourteen soybean cultivars (ten cultivated and four wild) in positive and negative ion modes.

No.	Identification	Formula	t*_R_* (min)	Calc. Mass *m/z* [M]	Observed Mass *m*/*z* [M ± H]^±1^	(±) Fragment Ions
1	Choline	C_5_H_13_NO	0.93	103.0997	104.1069	60.0806
2	Iditol	C_6_H_14_O_6_	0.94	182.0790	181.0717	
3	Malic acid	C_4_H_6_O_5_	0.97	134.0215	133.0142	
4	Citric acid	C_6_H_8_O_7_	0.98	192.0270	191.0197	
5	Furoic acid	C_5_H_4_O_3_	0.99	112.0161	111.0088	67.0187
6	Catechin	C_15_H_14_O_6_	1.00	290.0791	289.0718	245.0812, 203.0707, 187.0398, 137.0240, 109.0291
7	Glutamic acid	C_5_H_9_NO_4_	1.04	147.0531	146.0458	102.0556, 84.0451
8	Gluconic acid	C_6_H_12_O_7_	1.05	196.0583	195.0510	129.0189, 75.0085, 59.0137
9	Aspartic acid	C_4_H_7_NO_4_	1.06	133.0375	132.0302	115.0032, 88.0401
10	Histidine	C_6_H_9_N_3_O_2_	1.09	155.0695	156.0768	110.0703, 93.0439
11	Malic acid	C_4_H_6_O_5_	1.20	134.0215	133.0142	115.0033, 71.0136
12	Isoleucine	C_6_H_13_NO_2_	1.27	131.0946	132.1019	86.0957, 69.0693
13	Cynaroside	C_21_H_20_O_11_	1.31	448.1006	449.1079	68.997
14	UDP-*N*-acetylglucosamine *	C_17_H_27_N_3_O_17_P_2_	1.34	607.0818	606.0745	384.9831, 158.9254, 96.9692
15	Tryptophan	C_11_H_12_N_2_O_2_	1.36	204.0898	205.0971	188.0692, 146.0589, 118.0642
16	Phenylalanine	C_9_H_11_NO_2_	1.38	165.0789	166.0863	120.0803, 103.0538, 91.0539
17	Catechin *	C_15_H_14_O_6_	1.47	290.0791	289.0718	187.0398, 137.0240, 109.0291
18	Catechin analog *	C_15_H_14_O_6_	4.41	290.0789	289.0717	245.0812, 203.0707, 137.0240, 109.0291
19	3,5-Dihydroxy-2-(4-hydroxyphenyl)-4-oxo-3,4-dihydro-2H-chromen-7-yl hexopyranoside	C_21_H_22_O_11_	4.57	450.1162	449.1089	287.0554, 259.0604, 125.0240, 57.0345
20	Apigetrin	C_21_H_20_O_10_	4.91	432.1056	431.0983	269.0446, 117.0339, 89.0240
21	Daidzin	C_21_H_20_O_9_	5.82	416.1106	417.1179	227.0684, 199.0738, 137.0223
22	Daidzein	C_15_H_10_O_4_	6.26	254.0577	255.0650	225.0552, 208.0527, 113.0295, 91.0187, 65.0033
23	Apigetrin analog	C_21_H_20_O_10_	6.47	432.1054	477.1035	269.0446, 117.0339, 89.0240
24	Glycitein	C_16_H_12_O_5_	6.64	284.0684	283.0612	268.0368, 240.0421
25	Genistein	C_15_H_10_O_5_	7.15	270.0527	269.0454	225.0547, 181.0654
26	Daidzein analog	C_15_H_10_O_4_	8.28	254.0579	253.0506	209.0598, 133.0292, 91.0186, 65.0031
27	Naringenin	C_15_H_12_O_5_	9.26	272.0685	271.0612	151.0028, 119.0498, 93.0341, 65.0032
28	Genistein analog	C_15_H_10_O_5_	9.53	270.0528	269.0455	225.0547, 181.0654
29	Corchorifatty acid F	C_18_H_32_O_5_	9.61	328.2251	327.2178	229.1441, 211.13303, 171.1020, 85.0291, 57.0345
30	(15Z)-9,12,13-Trihydroxy-15-octadecenoic acid *	C_18_H_34_O_5_	10.08	330.2407	329.2334	171.1020, 139.1123, 99.0812
31	Soyasaponin I	C_48_H_78_O_18_	10.76	942.5182	941.5105	615.3881, 457.3671, 205.0709, 143.0345, 113.0241
32	(±)9-HpODE *	C_18_H_32_O_4_	12.12	312.2301	311.2228	275.2005, 183.0115, 79.9569
33	(+/−)9,10-dihydroxy-12Z-octadecenoic acid *	C_18_H_34_O_4_	13.00	314.2458	313.2385	277.2168, 201.1125, 171.1022
34	13(S)-HOTrE *	C_18_H_30_O_3_	13.95	294.2195	293.2123	195.1390, 95.9597, 79.9570

* Differentiating metabolites.

**Table 2:** Identification of metabolites using high-resolution mass spectrometry of water extract of fourteen soybean cultivars (ten cultivated and four wild) in positive and negative ion modes.

No.	Identification	Formula	t*_R_* (min)	Calc. Mass *m/z* [M]	Observed *m/z* [M ± H]^±1^	(±) Fragment Ions
1	Arginine	C_6_H_14_N_4_O_2_	0.83	174.1116	173.1044	156.0778, 131.0822, 114.0557
2	Histidine	C_6_H_9_N_3_O_2_	0.85	155.0695	156.0768	110.0703, 93.0439
3	Glutamic acid	C_5_H_9_NO_4_	0.93	147.0531	146.0458	ND
4	Glucose	C_6_H_12_O_6_	0.93	180.0634	179.0561	101.024289.0242, 71.01369, 59.0138
5	*N*-acetylornithine	C_7_H_14_N_2_O_3_	0.94	174.1004	173.0931	131.0822, 85.0769
6	D-Ribose	C_5_H_10_O_5_	0.95	150.0528	149.0455	131.0346, 89.0243, 71.0137, 59.0138
7	Malic acid	C_4_H_6_O_5_	0.97	134.0215	133.0142	115.0034, 89.0242, 72.9929, 71.0137
8	Glutamine *	C_5_H_10_N_2_O_3_	0.99	146.0692	147.0765	127.0509, 109.0404, 84.0452
9	Uridine	C_9_H_12_N_2_O_6_	1.00	244.0695	243.0623	200.0562, 152.0351, 110.0245
10	Citric acid	C_6_H_8_O_7_	1.00	192.0269	191.0196	173.0090, 111.0085, 87.0087
11	Arginine	C_6_H_14_N_4_O_2_	1.00	174.1117	175.1189	156.0778, 131.0822, 114.0557
12	Furoic acid	C_5_H_4_O_3_	1.01	112.0161	111.0088	67.0189
13	Pantothenic acid	C_9_H_17_NO_5_	1.01	219.1106	218.1033	146.0821, 88.0402
14	Threonine	C_4_H_9_NO_3_	1.02	119.0582	120.0655	116.0696, 70.0645
15	Asparagine	C_4_H_8_N_2_O_3_	1.03	132.0535	133.0608	116.0333, 87.0546, 74.0231
16	Proline	C_5_H_9_NO_2_	1.03	115.0633	116.0706	70.0645
17	Trans-Aconitic acid	C_6_H_6_O_6_	1.03	174.0164	173.0092	129.0191, 111.0085, 85.02936
18	Cytosine	C_4_H_5_N_3_O	1.04	111.0432	112.0505	95.0232, 69.0442
19	Glutamic acid	C_5_H_9_NO_4_	1.04	147.0532	148.0605	128.0348, 102.0556, 84.0451
20	Lysine	C_6_H_14_N_2_O_2_	1.04	146.1055	147.1128	130.0852, 84.0801, 56.0491
21	Valine	C_5_H_11_NO_2_	1.08	117.0789	118.0862	100.0748, 72.0802, 55.0538
22	Adenine	C_5_H_5_N_5_	1.11	135.0545	136.0617	119.0343
23	Guanine *	C_5_H_5_N_5_O	1.11	151.0494	152.0567	135.0294, 110.0341
24	Succinic acid	C_4_H_6_O_4_	1.13	118.0266	117.0193	73.0293
25	*N*-Acetylornithine	C_7_H_14_N_2_O_3_	1.21	174.1005	175.1077	131.0822, 85.0769
26	Guanosine	C_10_H_13_N_5_O_5_	1.21	283.0917	282.0844	150.0419, 108.0202
27	Hypoxanthine *	C_5_H_4_N_4_O	1.21	136.0385	137.0458	119.0342, 110.0340, 94.0392
28	Tyrosine *	C_9_H_11_NO_3_	1.22	181.0739	180.0666	163.0396, 119.0498, 72.0088
29	Methionine	C_5_H_11_NO_2_S	1.25	149.0511	150.0583	133.0307, 104.0520, 61.0102
30	Isoleucine	C_6_H_13_NO_2_	1.30	131.0946	132.1018	86.0957, 69.0693
31	Glutaric acid *	C_5_H_8_O_4_	1.32	132.0422	131.0349	113.0240, 87.0449, 69.0343
32	Leucylproline	C_11_H_20_N_2_O_3_	1.40	228.1473	229.1546	116.0696, 86.0957, 70.0645
33	Trans-3-Indoleacrylic acid	C_11_H_9_NO_2_	1.80	187.0632	188.0705	170.0586, 146.0588, 118.0641
34	Tryptophan	C_11_H_12_N_2_O_2_	1.80	204.0898	205.0971	188.0692, 146.0589, 118.0642
35	Glycidic acid	C_10_H_10_O_3_	2.17	178.0629	177.0557	133.0656, 71.01366
36	12-O-Î²-D-Glucopyranosyloxyjasmonic acid	C_18_H_28_O_9_	4.00	388.1732	387.1659	207.1023, 101.0242, 89.0242, 59.0138
37	Sinensin	C_21_H_22_O_11_	4.26	450.1162	449.1089	287.0555, 259.0605, 125.02401
38	Dihydrophaseic acid	C_15_H_22_O_5_	4.63	282.1468	281.1395	171.1175, 123.0813, 87.00853
39	12-O-Î²-D-Glucopyranosyloxyjasmonic acid	C_18_H_28_O_9_	4.95	388.1733	387.166	207.1023, 101.0242, 89.0242, 59.0138
40	Daidzin *	C_21_H_20_O_9_	5.30	416.1108	417.1181	227.0684, 199.0738, 137.0223
41	Glycitein	C_16_H_12_O_5_	5.33	284.0684	283.0611	268.0371, 240.0422
42	Hdroxycaproic acid *	C_6_H_12_O_3_	5.39	132.0786	131.0714	113.0606, 85.0656, 57.0345
43	*N*-Acetyl-l-phenylalanine	C_11_H_13_NO_3_	5.72	207.0895	206.0823	164.0713, 91.0551, 70.0296
44	Pheyllactic acid *	C_9_H_10_O_3_	5.99	166.0629	165.0557	147.0449, 119.0501, 72.9929
45	2-(acetylamino)-3-(1H-indol-3-yl)propanoic acid	C_13_H_14_N_2_O_3_	6.30	246.1004	245.0931	116.0349, 98.0244, 74.0245, 58.0297
46	Apigetrin *	C_21_H_20_O_10_	6.34	432.1056	431.0983	268.0368, 239.0335, 59.0137
47	Genistin *	C_21_H_20_O_10_	6.47	432.1057	433.1129	271.0579, 215.0685, 153.0170
48	Daidzein *	C_15_H_10_O_4_	6.48	254.0578	253.0505	208.0527, 113.0295, 91.0187, 65.0033
49	Astragalin	C_21_H_20_O_11_	6.51	448.1005	447.0932	284.0320, 227.0345, 65.0032
50	Glycitein analog	C_16_H_12_O_5_	6.56	284.0684	283.0611	268.0371, 240.0422
51	Octyl glucoside	C_14_H_28_O_6_	6.72	292.1886	291.1813	85.0292, 59.0137
52	7-Hydroxy-2-(4-hydroxyphenyl)-4-oxo-3,4-dihydro-2H-chromen-5-yl β-d-glucopyranoside *	C_21_H_22_O_10_	7.04	434.1213	433.1139	271.0607, 243.0667, 151.0034, 93.0343
53	Azelaic acid	C_9_H_16_O_4_	7.32	188.1048	187.0976	125.0969, 97.0656
54	Galangin *	C_15_H_10_O_5_	7.49	270.0527	271.0599	215.0686, 153.0169, 115.0532
55	Phloretin *	C_15_H_14_O_5_	7.77	274.0841	273.0768	167.0346, 123.0447, 93.0343
56	Abscisic acid	C_15_H_20_O_4_	8.01	264.1362	263.1288	219.1386, 204.2252, 136.0526
57	Daidzein analog	C_15_H_10_O_4_	8.18	254.0578	253.0505	225.0552, 132.0214, 91.0187, 65.0033
58	5,7-dihydroxy-3-(4-methoxyphenyl)-4H-chromen-4-one *	C_16_H_12_O_5_	8.56	284.0685	285.0757	229.0839, 197.0579, 118.0402
59	Genistein	C_15_H_10_O_5_	9.43	270.0526	269.0454	225.0557, 181.0654
60	(15Z)-9,12,13-trihydroxy-15-octadecenoic acid	C_18_H_34_O_5_	10.01	330.2405	329.2332	211.1335, 171.1023, 139.1125, 99.0813
61	Tetradecanedioic acid	C_14_H_26_O_4_	10.63	258.1831	257.1758	239.1644
62	Soyasaponin I	C_48_H_78_O_18_	10.77	942.5182	941.5107	733.4519, 457.3673, 257.0659, 101.0242
63	(±)9-HpODE	C_18_H_32_O_4_	11.34	312.2301	311.2228	171.1023, 139.1125, 113.0968
64	Thapsic acid	C_16_H_30_O_4_	11.75	286.2144	285.2071	267.2065, 59.0137

* Differentiating metabolites.

## Data Availability

Data sharing is not applicable to this article.
